# Screening of Wood/Forest and Vine By-Products as Sources of New Drugs for Sustainable Strategies to Control *Fusarium graminearum* and the Production of Mycotoxins

**DOI:** 10.3390/molecules26020405

**Published:** 2021-01-14

**Authors:** Mathilde Montibus, Xavier Vitrac, Véronique Coma, Anne Loron, Laetitia Pinson-Gadais, Nathalie Ferrer, Marie-Noëlle Verdal-Bonnin, Julien Gabaston, Pierre Waffo-Téguo, Florence Richard-Forget, Vessela Atanasova

**Affiliations:** 1 Institut Technologique FCBA, Allée de Boutaut, BP 227, F-33028 Bordeaux, France; mathilde.montibus@fcba.fr; 2 Laboratoire Phenobio SAS, ZA Les Pins Verts, 22 Allée de Migelane, F-33650 Saucats, France; xavier.vitrac@phenobio.fr; 3 Laboratoire de Chimie des Polymères Organiques, Université de Bordeaux, CNRS, Bordeaux INP, UMR 5629, 16 Avenue Pey-Berland, F-33600 Pessac, France; veronique.coma@u-bordeaux.fr (V.C.); anne.loron@enscbp.fr (A.L.); 4 INRAE, UR1264 Mycology and Food Safety (MycSA), F-33882 Villenave d’Ornon, France; laetitia.pinson-gadais@inrae.fr (L.P.-G.); nathalie.ferrer@inrae.fr (N.F.); marie-noelle.verdal-bonnin@inrae.fr (M.-N.V.-B.); florence.forget@inrae.fr (F.R.-F.); 5 Faculté des Sciences Pharmaceutiques, Unité de Recherche Œnologie, EA 4577, USC 1366 INRAE, Equipe Molécules d’Intérêt Biologique-ISVV, Université de Bordeaux, F-33882 Villenave d’Ornon, France; julien.gabaston@gmail.com (J.G.); pierre.waffo-teguo@u-bordeaux.fr (P.W.-T.)

**Keywords:** *Fusarium graminearum*, type B trichothecenes, natural extracts, ecological strategies, biofungicides

## Abstract

*Fusarium graminearum* is a fungal pathogen that can colonize small-grain cereals and maize and secrete type B trichothecene (TCTB) mycotoxins. The development of environmental-friendly strategies guaranteeing the safety of food and feed is a key challenge facing agriculture today. One of these strategies lies on the promising capacity of products issued from natural sources to counteract crop pests. In this work, the in vitro efficiency of sixteen extracts obtained from eight natural sources using subcritical water extraction at two temperatures was assessed against fungal growth and TCTB production by *F. graminearum*. Maritime pine sawdust extract was shown to be extremely efficient, leading to a significant inhibition of up to 89% of the fungal growth and up to 65% reduction of the mycotoxin production by *F. graminearum*. Liquid chromatography/mass spectrometry analysis of this active extract revealed the presence of three families of phenolics with a predominance of methylated compounds and suggested that the abundance of methylated structures, and therefore of hydrophobic compounds, could be a primary factor underpinning the activity of the maritime pine sawdust extract. Altogether, our data support that wood/forest by-products could be promising sources of bioactive compounds for controlling *F. graminearum* and its production of mycotoxins.

## 1. Introduction

*Fusarium graminearum* was recently ranked as the fourth most important fungal plant pathogen regarding scientific and economic criteria [1]. This fungus infests cereal crops and can produce type B trichothecenes (TCTB) mycotoxins that are of major concern due to their toxicity to animals and humans [2]. TCTB include deoxynivalenol (DON) and its acetylated forms 3-acetyl-4-deoxynivalenol and 15-acetyl-4-deoxynivalenol (3ADON and 15ADON), and nivalenol (NIV) and its acetylated form 4-acetylnivalenol or fusarenon X (FX). DON is the most commonly found TCTB on crops. DON is regulated in many countries worldwide, with a strict limitation set at 1750 µg kg^−1^ for unprocessed maize, durum wheat and oats in the European Union (European Commission in the Commission Regulation No 1126/2007), at 2000 µg kg^−1^ for unprocessed wheat in Canada and at 1000 µg kg^−1^ for cereals in China, for example [3].

Combined with good agricultural practices, the use of fungicides is a key factor in the integrated management strategies aiming to tackle this economical and public health issue. Their repeated use over decades has, however, disrupted natural biological systems and resulted in the development of fungal resistance [4]. In addition, the European regulation REACH and the French Ecophyto plans have been established to decrease the use of synthetic fungicides over the next few years. There is therefore a major need for bio-based and non-toxic alternatives, efficient to prevent *Fusarium* growth and its production of mycotoxins.

In the context of biopesticide formulations to control phytopathogenic fungi such as *F. graminearum*, a wide range of plant extracts has showed a broad spectrum of activity. Several of these extracts were characterized by a richness in terpenes [5,6] and phenolic compounds, for which antifungal properties have been widely described, including their capacity to reduce the mycelial growth of *F. graminearum* and its mycotoxin production [7,8,9]. Caffeic acid [10] or ferulic acid [11] have been the most extensively studied for their ability to inhibit the yield of TCTB while tetrahydrocurcumin was shown to minimize the accumulation of fumonisins produced by *Fusarium proliferatum* [12]. Compared to pure molecules, natural crude or partially purified extracts from plants could be of interest due to the potential benefit resulting from synergic effects between different active substances [13]. Another advantage is that a mixture of active molecules with different fungal physiological targets can limit the development of resistance. 

Forests and wood by-products are currently poorly valorized, although they could be promising sources of extracts characterized by a high abundance of phenolic compounds or terpenes. Thus, extracts of seven Amazonian woods including *Acacia mangium* (acacia), *Paraserianthes falcataria* (sengon) and *Swietenia mahagoni* (mahoni) barks have been shown to efficiently reduce wood rot diseases [14,15]. Similarly, the capacity of spruce bark extracts to inhibit the development of *Plasmopara viticola*, the causal agent of downy mildew on grapevine, has been reported [16]. Extracts from Norway spruce barks have been characterized for their potential to reduce the mycelial growth of different fungi as well as bacteria [17]. 

One of the guiding ideas of the present work was to exploit the richness in potential bioactive compounds of wood/forest and vine by-products and provide a use for this neglected biomass. Indeed, vineyard management often implies cane and vine-shoot pruning, leaf trimming and grub up vineyards and was reported to generate annually about 5 tons of solid waste per hectare [18]. In the same way, wood/forest wastes are a versatile raw material that can be used for various applications. According to a Parliament briefing, in 2012, 52.9 million tons of wood waste were produced in the European Union [19].

Driven by environmental issues, the objective of this work was to investigate the biological activity of natural extracts obtained from wood/forest and vine by-products using a subcritical water extraction procedure. After characterization of the total phenolic content and the antioxidant activity of each extract, their capacity to restrain the fungal growth of *F. graminearum* and to inhibit the production of TCTB were evaluated. Additionally, to better understand composition/bioactivity relationships, a deeper study of the phenolic composition of the most active extract was carried out using liquid chromatography/mass spectrometry. 

## 2. Results

### 2.1. Characterization of Wood/Forest and Vine By-Product Extracts

#### 2.1.1. Plant Extraction Yield

A subcritical water extraction method was used to produce sixteen extracts from eight wood/forest and vine by products. Subcritical water is defined as hot water at sufficient pressure to maintain the liquid state at a critical temperature between 100 °C (the boiling point of water) and 374 °C (the critical point of water) under the critical pressure (1–22.1 MPa). As the temperature increases, the dielectric constant of the water changes, so that the polar, medium polar, weakly polar, and nonpolar compounds can be extracted differentially. In this study, two temperatures, 125 °C and 175 °C, were used for the extraction, leading to extracts designed as extract 125 °C and extract 175 °C.

The obtained extraction yields associated with the different plant materials are presented in Table 1. Our results showed how the extraction yields varied with samples, from 1.1% for locust chips 125 °C, to 7.9% for maritime pine needles 175 °C. Extracts obtained from the same plant source but with different extraction conditions resulted in similar extraction yields with the exception of vine root extracts (extraction yield of vine roots 175 °C was 3.7 times higher than that of vine roots 125 °C). The highest extraction yields were observed for the two maritime pine needle extracts (7–8%), vine roots 175 °C (7%), the two vine cane extracts (5–6%), and the two maritime pine bark extracts (5%). Lower extraction yields, below 5%, were obtained for the two locust chip, chestnut chip, oak chip, maritime pine sawdust extracts and the vine root 125 °C extract.

#### 2.1.2. Total Phenolic Content and Antioxidant Activity of Wood/Forest and Vine By-Product Extracts

**Figure 1:**
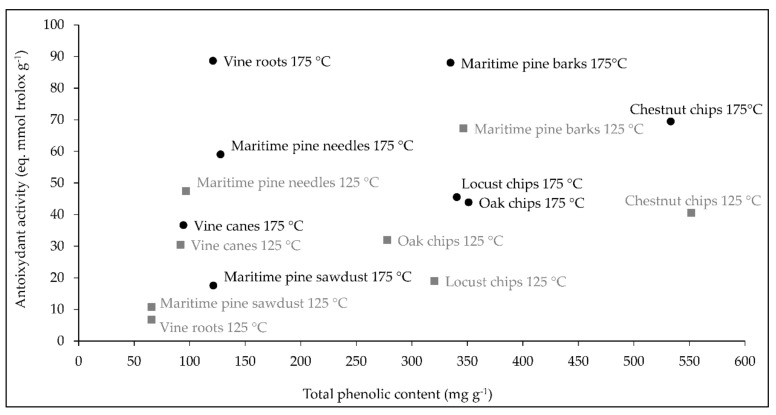
Correlation between total phenolic content and antioxidant activity of extracts obtained at 175 °C (black circles) and extracts obtained at 125 °C (gray squares). Data are means using three biological replicates.

The total phenolic content of the natural extracts considered in the present study are gathered in Table 1. The antioxidant activity values can be retrieved in Figure 1. According to their phenolic concentration, the extracts can be classified into three groups: 

Low total phenolic concentration (<200 mg g^−1^) for maritime pine sawdust, maritime pine needle, vine cane, and vine root extracts obtained at 125 and 175 °C.

Intermediate total phenolic concentration (ranging from 200 to 400 mg g^−1^) for maritime pine bark, oak chip, and locust chip extracts obtained at 125 and 175 °C.

High total phenolic concentration (>500 mg g^−1^) for chestnut chip extracts obtained at 125 and 175 °C.

Overall, total phenolic concentration and antioxidant activity of extracts obtained from the same plant source at the two temperatures were quite similar, with the exception of vine root and locust chip extracts where the antioxidant activity of the extracts 175 °C was 13 and 2 times higher than that in the extracts 125 °C, respectively (Figure 1).

As shown in Figure 1, a significant positive correlation was observed between antioxidant activity and total phenolic content (r > 0.351, *p* < 0.015). However, for some extracts, such as vine roots 175 °C and maritime pine needles 175 °C, the high antioxidant activity (88.64 and 59.05 eq. mmol trolox g^−1^, respectively) was not associated with a high phenolic content.

### 2.2. Screening of the Wood/Forest and Vine By-Product Extracts for Their Antifungal Activity Against F. graminearum CBS 185.32

**Figure 2:**
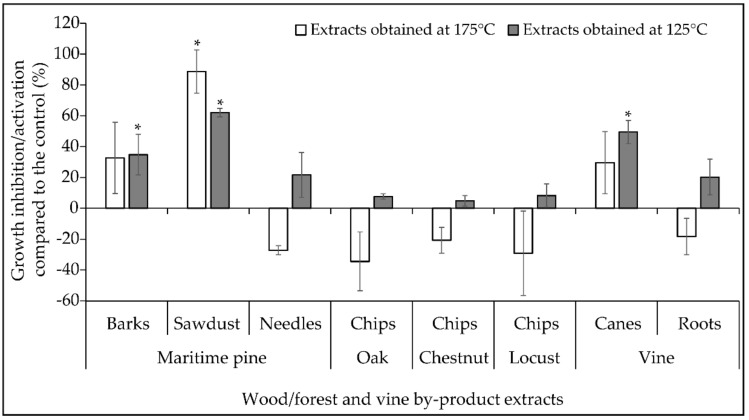
Percentage of inhibition/activation of the radial growth of *Fusarium graminearum* by the natural extracts obtained at 175 °C (white bars) and extracts obtained at 125 °C (gray bars). Data are means ± standard deviation using three biological replicates. Asterix (*) indicates significant differences when compared with control (Student’s t test, control versus treated, *p* < 0.05). The inhibitory activity of extracts was calculated according to the following formula: percentage of mycelial growth inhibition % = [(area in control culture − area in extract-amended culture)/area in control culture] × 100.

A first screening assay was implemented to assess whether the wood/forest and vine by-products considered in the present study could be promising sources of subcritical water extractable antifungal compounds. The effect of crude extracts obtained from the same initial quantity of raw materials on the radial growth of *F. graminearum* CBS 185.32 strain was investigated in Potato Dextrose Agar (PDA) medium. The extract activities were expressed as the percentage of growth inhibition/activation compared to the corresponding control (Figure 2). Our data indicated that the maritime pine sawdust 175 °C, maritime pine sawdust 125 °C, vine cane 125 °C, and maritime pine bark 125 °C extracts led to a significant inhibition of the fungal growth with inhibition percentages ranging between 35 and 89%. The highest inhibition rate was obtained for the maritime pine sawdust source and the 175 °C extraction procedure. None of the other extracts had any statistically significant effect on the fungal growth. Indeed, the inhibitions observed in PDA media with the maritime pine bark 125 °C, vines cane 125 °C, maritime pine needle 175 °C and vine root 175 °C extracts, ranging between 20–33%, were not statistically significant. In contrast, several extracts including oak chips 175 °C, locust chips 175 °C and maritime pine needles 175 °C were shown to increase non-significantly the fungal growth by 34, 29, and 27% compared to the control, respectively.

No correlation was evidenced between the antifungal activity of the extracts and the extract yield, neither with the antioxidant activity nor the total phenolic content. 

We further investigated the effect of the most promising extracts, i.e., maritime pine sawdust 175 °C, maritime pine sawdust 125 °C, vines cane 125 °C, and maritime pine bark 125 °C extracts, on the production of TCTB by *F. graminearum* CBS 185.32.

### 2.3. Impact of Selected Wood/Forest and Vine By-Product Extracts on the Mycelial Biomass of F. graminearum and the Production of TCTB in Liquid Cultures

#### 2.3.1. Comparative Efficiencies of Maritime Pine Sawdust 175 °C, Maritime Pine Sawdust 125 °C, Vine Cane 125 °C and Maritime Pine Bark 125 °C Extracts to Affect the Fungal Growth and the Production of TCTB by F. graminearum CBS 185.32

**Figure 3:**
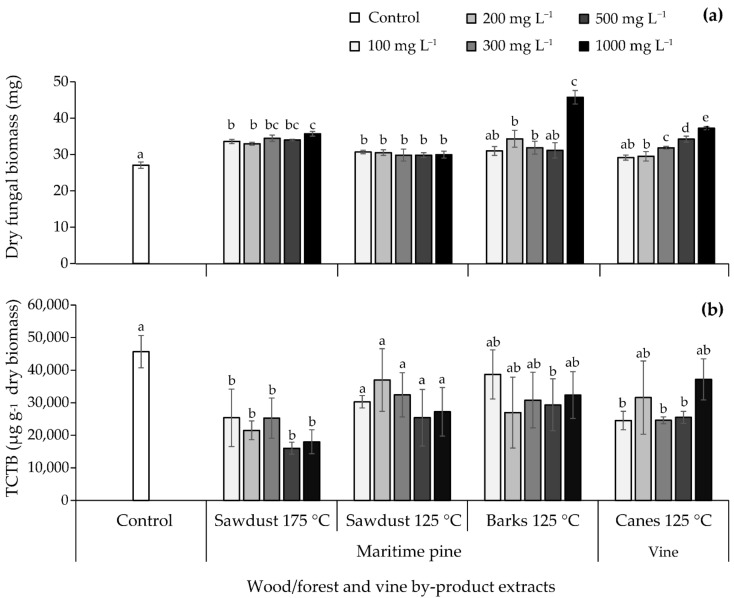
Effect of natural extracts obtained at 125 °C or 175 °C at 100, 200, 300, 500, and 1000 mg L^−1^ on (**a**) fungal biomass and (**b**) type B trichothecenes (TCTB) production by *Fusarium graminearum* CBS 185.32. Values are expressed as means ± standard deviation using four biological replicates. Differences between control and tested extract concentrations in terms of fungal biomass and TCTB were determined separately for each extract with multiple comparisons using the Dunn–Sidak method. For each extract, different letters indicate significant differences (*p* < 0.05).

The effect of the maritime pine sawdust 175 °C, maritime pine sawdust 125 °C, vine cane 125 °C and maritime pine bark 125 °C extracts on TCTB production by *F. graminearum* CBS 185.32 was studied in the appropriate toxin-inducing liquid Minimal Synthetic medium (MS) [8,10]. Each natural extract was tested at five concentrations: 100, 200, 300, 500, and 1000 mg L^−1^; results are gathered in Figure 3a,b.

With the exception of maritime pine bark 125 °C extract at 100 and 500 mg L^−1^ and vine cane 125 °C extract at 100 mg L^−1^, the different extracts induced a significant increase in the biomass ranging between 9 and 69% (Figure 3a). The highest activation rate (69%) was observed for the maritime pine bark 125 °C extract at 1000 mg L^−1^.

DON and 15ADON were the major TCTB produced by the *F. graminearum* CBS185.32 strain in MS medium. Because the DON/15ADON ratio was not significantly affected by the extracts, the sum of the two mycotoxins was used to express the amount of TCTB produced.

The maritime pine bark 125 °C and maritime pine sawdust 125 °C extracts at all the tested concentrations did not affect the TCTB production, with the exception of maritime pine barks 125 °C at 500 mg L^−1^ that induced a significant inhibition of 36%. The supplementation of liquid cultures with vine cane 125 °C extract at 100, 300, and 500 mg L^−1^ led to a significant reduction close to 46% in TCTB accumulation. The maritime pine sawdust 175 °C extract at all the tested concentrations was characterized by the highest potential to reduce the TCTB production; inhibitions of 53, 65, and 61% were observed with 200, 500, and 1000 mg L^−1^, respectively.

Overall, our data indicated that among the four extracts selected for their capacity to restrain the radial fungal growth in PDA medium, the maritime pine sawdust 175 °C extract was the most effective to reduce the TCTB yield with an inhibition reaching more than 50%. The 200 and 500 mg L^−1^ concentrations were selected for the next step of our work.

#### 2.3.2. Effect of the Maritime Pine Sawdust 175 °C Extract on the Fungal Biomass and TCTB Yield by a Panel of *F. graminearum* Strains

**Figure 4:**
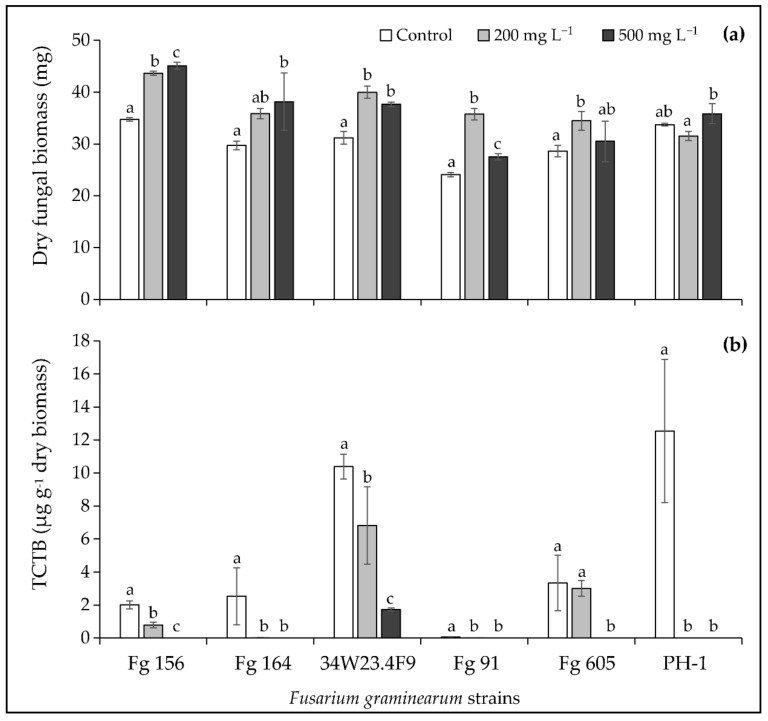
Effect of the maritime pine sawdust extract obtained at 175 °C at 200 and 500 mg L^−1^ on (**a**) fungal biomass and (**b**) type B trichothecenes (TCTB) production by six *Fusarium graminearum* (Fg) strains. Values are expressed as means ± standard deviation using four biological replicates. Differences between control and treated conditions in terms of fungal biomass and TCTB were determined separately for each fungal strain with multiple comparisons and the Dunn–Sidak method. For each fungal strain, different letters indicate significant differences between tested conditions (*p* < 0.05).

To confirm the promising potential of the maritime pine sawdust 175 °C extract, its effect on the TCTB production was investigated in liquid MS medium using six additional strains of *F. graminearum*. Results on the fungal biomass and the TCTB accumulation are reported in Figure 4a,b, respectively.

Whatever the *F. graminearum* strain, the maritime pine sawdust 175 °C extract at the two tested concentrations had no effect or significantly activated the fungal biomass, which corroborates the results obtained with the *F. graminearum* CBS 185.32 strain (Figure 4a).

As it clearly appears in Figure 4b, the TCTB amounts yielded by the six studied strains were significantly reduced by the treatments with the maritime pine sawdust 175 °C extract. At 500 mg L^−1^, the extract led to a complete inhibition of the mycotoxin accumulation by Fg 156, Fg 164, Fg 91, Fg 605, and PH-1, and induced an inhibition of 83% for the 34W23.4F9 strain. Whereas the TCTB yield was abrogated with 200 mg L^−1^ for the PH-1 strain, the decrease in TCTB accumulation was less drastic for the other strains (99, 61, 56 and 34% for Fg 164, Fg 156, Fg 91 and 34W23.4F9, respectively). Besides, no significant inhibition of TCTB production was observed for the Fg 605 strain in the broths supplemented with 200 mg L^−1^ of maritime pine sawdust 175 °C extract.

In conclusion, the maritime pine sawdust 175 °C extract can substantially inhibit the TCTB biosynthesis by *F. graminearum*, with an inhibition efficiency that varies according to the considered strain.

### 2.4. Characterization of the Phenolic Composition of the Maritime Pine Sawdust 175 °C Extract

**Figure 5:**
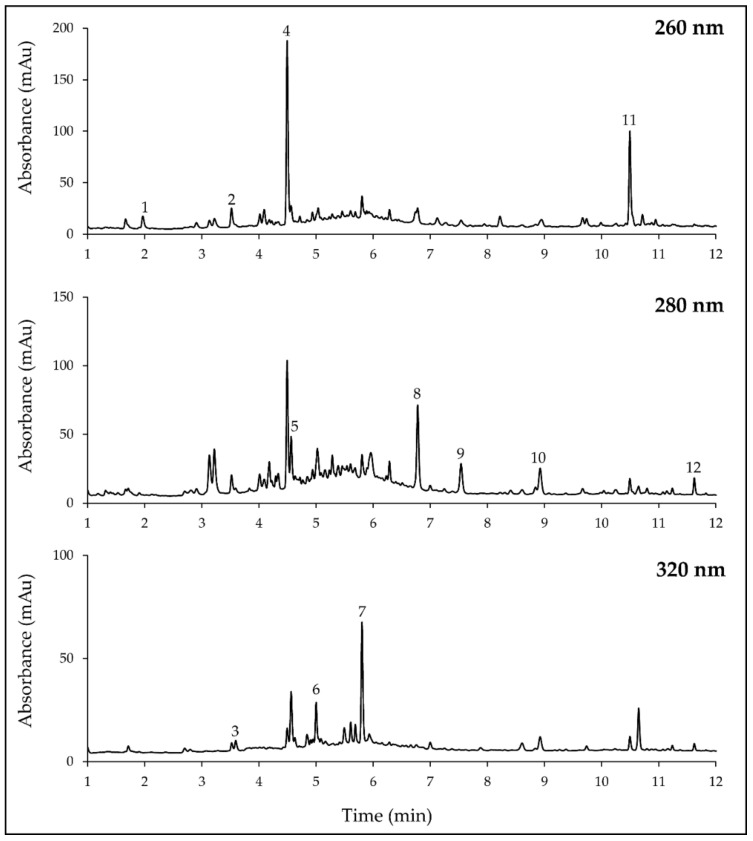
Chromatograms at 260, 280, and 320 nm of the active maritime pine sawdust extract obtained at 175 °C. 1, protocatechuic acid; 2, vanillic acid; 3, caffeic acid; 4, coniferyl alcohol; 5, vanillin; 6, ferulic acid; 7, coniferyl aldehyde; 8, nortrachelogenin; 9, pinoresinol; 10, pinobanksin; 11, unknown; 12, pinocembrin.

The soluble phenolic composition of the maritime pine sawdust 175 °C extract was characterized using liquid chromatography/diode-array detector coupled with mass spectrometry (LC-DAD/MS). Eleven peaks characterized by a high intensity were unequivocally identified by comparison with retention times, mass and UV-visible spectra of reference standards and quantified using their respective external calibration curves (Table 2, Figure 5). According to identification data, phenolic compounds were shown to belong to three main groups: phenolic acids/aldehydes/alcohols, lignans, and flavonoids.

The most representative group was the phenolic acids/aldehydes/alcohols with seven identified molecules (Table 2). Regarding the concentration values, coniferyl alcohol was the predominant compound with a concentration reaching 4.89 mg g^−1^, followed by coniferyl aldehyde, vanillin, vanillic acid, ferulic acid, protocatechuic acid, and caffeic acid. One additional peak eluted at the end of the chromatogram (10.5 min) was characterized by a UV-visible spectrum with λmax at 253 and 300 nm, i.e., with a slight hypsochromic effect in comparison with coniferyl alcohol (λmax at 264 and 300 nm), suggesting that this unidentified compound could belong to the same family. The compound eluting in this peak presented a deprotonated molecular ion at *m*/*z* 329. As we could not identify this compound, its concentration was estimated to be the equivalent of coniferyl alcohol (1.90 mg g^−1^).

The highest concentrations of phenolic compounds were measured in the group of lignans. Two lignans were identified and quantified in the active fraction: nortrachelogenin and pinoresinol at 7.30 mg g^−1^ and 2.84 mg g^−1^, respectively. The nortrachelogenin was the most abundant identified compound in the active extract.

Among the flavonoids, two compounds, the pinobanksin and its precursor (the pinocembrin), were identified and quantified at 1.01 and 0.41 mg g^−1^, respectively. 

Regarding the chemical structure of identified molecules, it is interesting to note that seven (coniferyl alcohol, coniferyl aldehyde, vanillin, vanillic acid, ferulic acid, nortrachelogenin, and pinoresinol) of the eleven identified compounds were mono- or di-methylated. The sum of the concentrations of methylated compounds was 17.38 mg g^−1^, which represents 82% of the quantified compounds. 

To summarize, 11 compounds belonging to three different groups of phenolics were unequivocally identified in the active maritime pine sawdust 175 °C extract. The group of phenolic acids contained the largest number of molecules and was estimated to represent 0.95% (*w w*^−1^) of the dried biomass of the extract. The main compound in this group was coniferyl alcohol representing 0.49% (*w w*^−1^) of the dried biomass. The group of lignans was represented by two compounds: nortrachelogenin and pinoresinol, contributed to 1.01% (*w w*^−1^) of the dried biomass. The lowest phenolic concentrations were associated with flavonoids (about 0.14% (*w w*^−1^)). The mono- and di-methylated phenolic molecules were shown to predominate qualitatively and quantitatively in the active extract.

## 3. Discussion

The aim of this study was to characterize natural extracts obtained from wood/forest and vine by-products using an environment-friendly extraction procedure for their capacity to reduce *F. graminearum* growth and TCTB accumulation, and to provide first insights regarding the compounds involved in their bioactivity.

### 3.1. Variations in Antioxidant Activity and Total Phenolic Composition of Wood/Forest and Vine By-Product Extracts

In the first part of this work, sixteen extracts were obtained from eight natural sources using a dynamic subcritical water extraction method and two experimental conditions, 125 and 175 °C. Characterization of the total phenolic composition and antioxidant activity of these extracts led to values ranging from 65.6 to 551.6 mg g^−1^ and from 6.7 to 88 eq. mmol trolox g^−1^, respectively. Concerning total phenolic composition, values associated with maritime pine barks (more than 300 mg g^−1^) were higher than the results previously reported for barks, between 22 and 62 mg g^−1^ depending on the extraction method [20]. Regarding the chestnut source, our results were fully consistent with previous data indicating a total phenolic content of approximatively 500 mg g^−1^ [21,22]. Concerning the antioxidant activity, the highest values (ranging from 66 to 88 eq. mmol Trolox g^−1^) were obtained for chestnut chip 175 °C, maritime pine bark 125 °C, maritime pine bark 175 °C and vine root 175 °C extracts. For pine bark extracts, antioxidant potential was higher than that obtained by Legault at al. [23] for different bark extracts using the same oxygen radical absorbance capacity method (ORAC). However, the comparison of our data regarding total phenolic composition and the antioxidant activity with previously published results have to be considered with caution as these values are dependent on method, condition, and origin of the raw material.

In the present study, the antioxidant activity of the extracts was positively correlated with total phenolic contents and seemed to be influenced by phenolic composition. The same trend has frequently been reported in previous studies focusing on natural extracts [24,25]. The high levels of antioxidant activity of chestnut chip 175 °C, maritime pine bark 125 °C, and maritime pine bark 175 °C extracts could be related to their phenolic composition. Maritime pine bark extracts are acknowledged to contain high levels of procyanidins (condensed tannins) [20], whereas chestnut chip extracts have been reported to contain high amounts of both procyanidins and ellagitannins (hydrolysable tannins) [26]. All these extract constituents are polymerized structures containing many hydroxyl functional groups which are supposed to contribute to the antioxidant activity of the extracts. Indeed, Yokozawa et al. [27] postulated that an increase in molecular weight, number of hydroxyl and galloyl groups, and ortho-hydroxyl structure improved the antioxidant activity of molecules, being important features for the scavenging of free radicals. On the contrary, when the free hydroxyl group was methoxylated or glycosylated, the antioxidant activity was decreased or even abolished [27]. Accordingly, the lower antioxidant activity of the active maritime pine sawdust 175 °C extract could be explained, on the one hand, by the presence of a multitude of methylated compounds at high amounts, and on the other hand, by its relatively low concentrations in total phenolics (121.3 mg g^−1^). 

However, in the present study, several extracts including maritime pine needle 175 °C and vine root 175 °C extracts were characterized by a high antioxidant activity but a low total phenolic content. The latter finding could be explained by the fact that the presence of other non-phenolic compounds could contribute to the overall antioxidant potential. Pine extracts could contain some lipophilic metabolites such as terpenoids and resin acids that certainly contribute to the antioxidant activity [28,29].

### 3.2. The Maritime Pine Sawdust 175 °C Extract Is a Strong Inhibitor of Fungal Growth and TCTB Production by F. graminearum CBS 185.32

Among the sixteen natural extracts studied in the present work, the two maritime pine sawdust, the vine cane 125 °C and the maritime pine bark 125 °C extracts were shown as the most promising for their capacity to restrain the *F. graminearum* CBS 185.32 radial growth. Several studies have previously highlighted the antifungal or antibacterial activity of extracts obtained from various parts of the pine tree [16,30,31,32]. However, to our knowledge, the present work is the first report demonstrating the antifungal activity of maritime pine sawdust extract against *F. graminearum*. For example, extracts from Palestian Aleppo pine seeds, barks, and cones were reported to reduce by 80–95% the growth of *Shigella*, *Escherichia coli* and *Staphylococcus aureus* [32]. In the study of Jung et al. [31], the effect of pine needle extracts was shown against several fungi and furfural was demonstrated as the active molecule responsible for the growth inhibition of *Alternia mali*. Spruce bark extract, which is considered as a wood industry by-product, was also identified as a potential inhibitor of downy mildew (*Plasmopara viticola*) [16]. While the antimicrobial activity of pine extracts has been the subject of several studies, to our knowledge, their effect on mycotoxin production by different mycotoxigenic fungi has never been reported previously. For the first time, the present study evidenced the capacity of pine sawdust extract 175 °C to efficiently limit the production of TCTB by a panel of *F. graminearum* strains.

Several hypotheses can be raised to explain the activities of the pine sawdust 175 °C extract. Numerous studies have shown positive relations between antifungal efficiency of natural extracts, contents in total phenolics and antioxidant potential, i.e., free radical scavenging capacity [33,34]. In addition, several authors have suggested that the efficiency of phenolic acids in reducing TCTB production could be linked to their antioxidant properties [7,35,36]. However, in the present study, no correlation was found between the antifungal effect of plant extracts, their total phenolic contents and antioxidant activities. Such an outcome was previously observed for 22 extracts from African tropical wood species [37]. The previous results suggest that in addition to the free radical scavenging activity, other mechanisms could explain the bioactivity of the extract. In accordance with the literature data [38], the capacity of natural compounds to disrupt the membrane integrity, perturb the ionic homeostasis and/or to directly interact with key fungal enzymes are additional properties that could explain their effect. Besides, lipophilic properties of natural extracts/compounds have been shown as primary factors sustaining the perturbations they can induce to fungal membranes. 

We have demonstrated that the active pine sawdust 175 °C extract was characterized by a qualitative and quantitative predominance of mono- and di-methylated phenolic compounds. Previous studies indicated that the main classes of natural antimicrobial phenolic compounds were methylated phenolics [39,40]. For example, 5,6,7,8-tetramethoxyflavone and 4′-hydroxy-5,6,7,8-tetramethoxyflavone characterized by four methoxyl groups were active against *Candida albicans* and *Staphylococcus aureus* [40]. In the study of Fitzgerald et al. [39], the structure-antifungal activity analysis of vanillin and some structural analogues has demonstrated that 4-anisaldehyde (one methoxyl group) showed lower Minimal Inhibitory Concentration (MIC) values than 4-hydroxybenzaldehyde (one hydroxyl group). Similarly, Koh et al. [41] have reported that pterostilbene (two methoxyl and one hydroxyl groups), but not resveratrol (three hydroxyl groups), detained potent fungicidal and sporicidal activities against *Leptosphaeria maculans* and could protect canola seedlings from blackleg infection. Recent studies, dealing with the efficiency of hydroxycinnamic acids to inhibit fungal growth and toxin biosynthesis by *Fusarium avenaceum, Aspergillus westerdijkiae* and *Penicillium verrucosum*, supported the key importance of methoxyl groups [42,43]. Since it is widely acknowledged that substitution with methoxyl groups contributes to the lipophilicity of a compound, we can suppose that the predominance of mono- and di-methylated phenolics in the maritime pine 175 °C extract partially explains its high efficiency.

The analysis of the phenolic composition of the maritime pine sawdust 175 °C extract has evidenced the occurrence of some phenolic compounds in which antifungal efficiency has been previously illustrated. According to Peng et al. [44], the pinocembrin flavonoid is efficient to control the growth of *Penicillium italicum* by interfering with energy homeostasis and inducing cell membrane damage in the pathogen. Nortrachelogenin has also been described as an efficient antifungal agent against *Candida albicans* [45]. According to the former study, high concentrations of nortrachelogenin were shown to induce membrane disruption, whereas low concentrations were responsible for a mitochondria and metacaspase-dependent apoptotic mechanism. In addition, by studying structure-activity relationships of phenolic acids, Shalaby et al. [46] postulated that the highest toxicity of ferulic acid (one methoxyl group) could be explained by its limited metabolization by *Cochliobolus heterostrophus*. The previous authors indicated that the lowest toxicity of coumaric and caffeic acids (one and two hydroxyl groups, respectively) could be related to their capacity to induce the expression of fungal genes encoding phenolic acid degrading enzymes. Besides, the afore published studies suggested that the inhibition of the trichothecene and enniatin mycotoxin production by ferulic acid could be explained by the reduction of the expression of some key biosynthetic genes suggesting the occurrence of a transcriptional control exerted by phenolic acid [9,11,42].

Finally, the antifungal and anti-mycotoxin activities of maritime pine sawdust 175 °C extract can certainly not be ascribed to the action of a single active molecule, but to the result of synergistic interactions between several compounds. Besides, other molecules, not identified in this study, in synergy or not with phenolics, certainly contribute to the bioactivity of the extract.

## 4. Materials and Methods 

### 4.1. Chemicals and Standards

For LC/MS analysis, LC-MS-grade acetonitrile from VWR (Fontenay-Sous-Bois, France) and formic acid from Fisher Scientific (Loughborough, U.K.) were purchased while water was purified by an Elga water purification system (High Wycombe, U.K.). Reagents were purchased from Scharlau (Barcelona, Spain), Sigma Aldrich (St Louis, MO, USA), and Fischer Scientific (Loughborough, U.K.).

Vanillic acid, protocatechuic acid, caffeic acid, ferulic acid, gallic acid, vanillin, coniferyl aldehyde, and pinocembrin were purchased from Sigma-Aldrich (St Louis, MO, USA) and coniferyl alcohol was obtained from Extrasynthese (Genay, France). Nortrachelogenin was isolated and purified from pine extract in the Molécules d’Intérêt Biologique laboratory (ISVV, Villenave d’Ornon, France) [47].

Standard solution of TCTB were purchased from Romer Labs (Tulln, Austria).

### 4.2. Natural Sources and Plant Material Preparation Prior to Extraction

The eight natural sources used in this study were collected in the Nouvelle Aquitaine Région, France in 2017. All sources came from agricultural by-products (vine canes and vine roots), and wood/forest by-products (maritime pine needles, barks and sawdust; oak chips; chestnut chips; and locust chips). The ethnobotanical data of used plant species are summarized in Table 1. Wood/forest by-products were supplied by the LESBATS society and vine by-products were collected by the Laboratory PHENOBIO. Upon receipt, the natural sources were dried at 40 °C during 7–10 days, ground into fine powder to pass through a 0.5 mm sieve, and stored at −20 °C.

### 4.3. Preparation of Natural Extracts and Determination of Extraction Yield and Extract Concentration

Dynamic subcritical water device used in this study included a High Performance Liquid Chromatography (HPLC) pump, a 300 mL extraction vessel, a heating device, a pressure controller, and a collector. For extraction of each raw material, 50 g was loaded into the high-pressure vessel. The vessel was placed in an oven set at 120 °C, and water was heated with a heating jacket at the chosen temperature (125 °C or 175 °C). The outlet valve of the extraction vessel was then closed and the system was pressurized to the desired pressure of 15 × 10^5^ Pa at a constant flow rate of 15 mL min^−1^. The liquid-to-solid ratio was maintained at the value of 5, to obtain 250 mL of extract. After the extraction vessel, the extract passed through a cooling system, was collected in a sampling vessel and then stored at −20 °C for further analysis.

The concentration of natural extracts was determined by lyophilizing an aliquote of 50 mL of each extract and weighing a dry sample. Extract concentration was expressed as a gram of dry matter per liter of extract. Extraction yield was calculated using the following formula: extraction yield % = (weight of extracted plant/weight of plant raw sample) × 100.

### 4.4. Fusarium Strains, Media, and Culture Conditions

Seven *F. graminearum* strains were used in this study; their origin and characteristics are summarized in Table 3.

When inoculum was required, fungal strains were grown on PDA at 25 °C for one week in the dark. Spore suspensions were prepared in carboxymethyl cellulose medium (CMC) (15 g L^−1^ carboxymethyl cellulose, 1 g L^−1^ yeast extract, 0.5 g L^−1^ MgSO_4_.7H_2_O, 1 g L^−1^ NH_4_NO_3_, 1 g L^−1^ KH_2_PO_4_). Four agar blocks (5 mm in diameter) carrying mycelia were introduced into 20 mL of CMC medium and incubated in darkness at 25 °C and at 180 rpm in a Multitron incubator shaker (INFORS AG, Bottmingen, Switzerland). After 2–3 days, culture broths were filtered in order to remove the mycelia. Spores were collected after centrifugation (2300× *g* for 10 min) and washed two times with sterile distilled water. Spores were suspended in 2% tween (for experimentations in PDA medium) or in MS medium (for experimentation in liquid MS medium), and concentration was determined using a spores counting method.

To screen the natural extracts at their initial concentration (Table 1) for their antifungal activity, a solid PDA medium and the CBS 185.32 strain were used. One milliliter of each natural extract was mixed with one milliliter of spore suspension at 10^4^ spores mL^−1^. Three times 20 µL of each mixture were equidistantly placed in a Petri dich (90 mm diameter). Three replications were performed for each individual treatment. Appropriate positive (Amphotericin B at 250 mg L^−1^) and negative controls were done. Cultures were incubated in the dark for 4 days at 25 °C. The extent of *F. graminearum* growth on each sample was photographed at day 2 and 3, and the fungal surface area was determined using the ImageJ software package developed by the National Institutes of Health (Bethesda, Maryland, USA). The inhibitory activity of extracts was calculated according to the following formula: percentage of mycelial growth inhibition % = [(area in control culture − area in extract-amended culture)/area in control culture] × 100.

Liquid-culture tests were done in MS medium as described by Gauthier at al. [8] Medium was supplemented or not with natural extracts at 100, 200, 300, 500, and 1000 mg L^−1^ depending of experimentation. Petri plates containing 8 mL of MS medium were inoculated with 2 × 10^4^ spores mL^−1^ and incubated in darkness at 25 °C for 10 days. Cultures were stopped by centrifugation (3000× *g* for 10 min) and 4 mL of each supernatant were stored at −20 °C before TCTB analysis. Fungal biomass production was measured by weighing the mycelia after 48 h of freeze-drying. For each trial, it was ensured that supplementation with extracts did not modify the pH values of the treated batches compared to controls. Four replications were done for each condition.

### 4.5. Extraction and TCTB Analysis

TCTB were extracted as described by Gauthier et al. [8] Quantification of mycotoxins was performed on a 1100 Series HPLC chain, equipped with an autosampler, an Agilent photodiode array detector and the ChemStation software (Agilent, Les Ulis, France). Separation was achieved on a Kinetex XB-C18 − 100 Å column (150 × 4.6 mm; 2.6 µm) (Phenomenex, Le Pecq, France) maintained at 45 °C. Mobile phase consisted of water acidified with *ortho*-phosphoric acid at pH 2.6 (solvent A) and acetonitrile (solvent B). TCTB were separated using a gradient elution as follows: 7 to 30% B in 10 min, 30–90% B in 5 min, 90% B for 5 min, 90 to 7% B for 2 min, 7% B for 5 min. The flow was kept at 1 mL min^−1^. The injection volume was 5 μL. The UV-visible spectra were recorded from 190 to 400 nm and peak areas were measured at 230 nm. Quantification was performed by external calibration. Toxin yields were expressed in μg g^−1^ of dry biomass.

### 4.6. Determination of Total Phenolic Content

The total phenolic content was spectrophotometrically measured according to 96-well microplates modified with the Folin–Ciocalteu method as previously described [49]. A microplate spectrophotometer (BMG Labtech, Champigny sur Marne, France) was used for measurement. Each well was filled with 20 μL of distilled water or natural extracts, followed by 100 μL of the Folin–Ciocalteu commercial solution diluted to 1/10 in water (Sigma-Aldrich, St Quentin Fallavier, France) and 80 μL of 7.5% (*w*/*v*) Na_2_CO_3_ solution. Prior to measuring the absorbance at 760 nm, the mixture was incubated for 20 min under dark conditions at room temperature. Gallic acid (0.03–0.5 mg L^−1^) was used as standard for calibration. The results were given as the mean of three determinations.

### 4.7. Determination of Free Radical Scavenging Potential by the Oxygen Radical Absorbance Capacity (ORAC)

The oxygen radical absorbance capacity analysis was carried out using 96-well fluorescence microplates and a FLUOstar Optima microplate reader (BMG LabTech, Champigny sur Marne, France). The reaction was carried out in a phosphate buffer (75 mM, pH 7.4); 30 μL of the extract solution, 180 μL of fluorescein (117 nM final concentration), and 90 μL of dihydrochloride (AAPH) at 40 mM were successively added to each well. The mixture was shaken and left to stand for 1.5 h at 37 °C. Fluorescence was recorded every minute during this period at excitation and emission wavelengths of 485 and 530 nm respectively. A blank sample (phosphate buffer replaced the sample) and Trolox^®^ calibration solutions (1–40 μM) were also performed simultaneously on the same microplate. The area under the curve (AUC) was calculated for each extract sample by integrating its relative fluorescence curve. By subtracting the AUC of the blank, the net AUC of the extracts was calculated and correlated with Trolox^®^ concentrations.

### 4.8. LC/MS Analysis

Analysis were performed using a 1290 Series UHPLC from Agilent Technologies (Santa Clara, CA, USA) equipped with an auto sampler module, a binary pump with degasser, a column heater/selector and an UV-visible diode-array detector. The UHPLC apparatus was coupled to an Esquire 6000 ion trap mass spectrometer using an electrospray ionization source (Bruker Daltonics, Billerica, MA, USA). 

Chromatographic elution was carried out on a 100 mm × 2.1 mm i.d., 1.8 µm, Zorbax SB-C18 column, with a 2.1 mm × 5 mm i.d. guard column (Agilent Technologies, Santa Clara, CA, USA). A binary solvent system composed of water acidified with 0.1% formic acid (solvent A) and acetonitrile acidified with 0.1% formic acid (solvent B) was used at a flow rate of 0.4 mL min^−1^ with the following gradient: 0–1.7 min, 10% B; 1.7–5.1 min, 10–30% B; 5.1–6.8 min, 30% B; 6.8–8.5 min, 30–35% B; 8.5–11.9 min, 35–60% B; 11.9–15.3 min, 60–100% B; 15.3–17 min, 100% B; 17–17.3 min, 100–10% B. Parameters of mass spectrometry were set in negative mode with a range of *m*/*z* 100−1200 with nitrogen as the drying gas at 10 L min^−1^, nebulizer pressure of 40 psi at 365 °C, capillary voltage at 3100 V, capillary exit voltage at −118.3 V, skimmer voltage at −40 V, and the trap drive at 58.1.

Maritime pine sawdust 175 °C extract was dissolved in methanol/water (3:7, *v*/*v*) at 10 mg mL^−1^, filtered on 0.45 µm and injected at 1 µL. The phenolic compounds were characterized by using commercial standards and the polyphenol library developed in Molécules d’Intérêt Biologique laboratory (ISVV, Villenave d’Ornon, France) [47,50]. Identification was performed by comparing retention time, UV data (λmax) as well as mass spectrometry data with pseudomolecular ion and product ions. Quantification was carried out for each compound with its own calibration curve at the maximum wavelength. Pure standards were injected at several concentrations (0, 1, 5, 20, 50, 100 μg mL^−1^) in independent triplicate to obtain calibration and equation curves. All data were processed with Bruker Data Analysis 3.2 software (Bruker Daltonics, Billerica, MA, USA).

### 4.9. Expression of Results and Statistical Analyses

Results of fungal growth and TCTB production were reported as mean values ± standard deviation of three or four biological replications depending of the experimentation.

The significance of the effect of the extracts on radial fungal growth in PDA medium was tested with Student’s t test (control versus treated).

For experimentations in liquid MS medium, one-way ANOVAs for TCTB and fungal biomass were carried out. Differences between control and tested extract concentrations in terms of fungal biomass and TCTB were determined separately for each extract or each strain with multiple comparison tests using the Dunn–Sidak method.

The Pearson correlation coefficient (r) was used to study the relationship between the antioxidant activity and the total phenolic content and between the antifungal efficiency of extracts and the total phenolic content, the antioxidant activity and the plant extraction yields.

The level of significance was set at *p* = 0.05.

Statistical analyses were performed using XLSTAT 2019 software (Addinsoft, Rennes, France).

## 5. Conclusions

In the present study, sixteen plant extracts were produced from eight wood/forest and vine by-products using a subcritical water extraction method, i.e., without the use of toxic solvents. On the one hand, the obtained findings showed that this green technology is appropriate for valorizing forest and agricultural by-products by producing extracts possessing various antioxidant properties and different levels of total phenolic content. Such extracts could be promising sources of bioactive molecules for different fields such as pharmaceutical, cosmetic, or food industries. On the other hand, our findings showed that several wood/forest and vine by-product extracts could be promising sources of subcritical water extractable inhibitors of *F. graminearum* radial growth. In addition, one extract from maritime pine sawdust was demonstrated to possess a high anti-mycotoxin capacity. This extract shows a great potential to be used for the development of biofungicide to combat *F. graminearum* and its TCTB production on agricultural commodities.

The phenolic characterization of the maritime pine sawdust 175 °C extract allowed us to make first hypotheses regarding its composition in active molecules and suggested that methylated compounds could be essential for its antifungal and anti-mycotoxin properties. However, it is most likely that biological activities are the result of a mixture of compounds rather than individual molecules, and that additive, synergistic, and/or antagonist effects between compounds of the same or different groups may be existing.

Further studies are necessary to determine the chemical identity of the active molecules responsible for the observed antifungal and anti-mycotoxin effectiveness. This essential step will allow providing the required basis to potentiate the properties of the maritime pine sawdust extract.

## Figures and Tables

**Table 1:** Origin, extraction yield, concentration, and total phenolic content of natural extracts.

Plant Species	Natural Extract	Extraction Yield (%) ^1^	Concentration (g L^−1^) ^2^	Total Phenolic Content (mg g^−1^)
Forest and Wood By-Products				
*Pinus pinaster*	Maritime pine barks 175 °C	5.0	10.0	334.8 ± 4.7
	Maritime pine barks 125 °C	4.7	9.5	346.6 ± 6.2
	Maritime pine sawdust 175 °C	2.3	4.6	121.3 ± 17.5
	Maritime pine sawdust 125 °C	1.8	3.7	65.8 ± 0.3
	Maritime pine needles 175 °C	7.9	15.9	127.8 ± 3.2
	Maritime pine needles 125 °C	7.3	14.6	96.7 ± 2.4
*Quercus robur*	Oak chips 175 °C	2.5	5.1	351.1 ± 11.1
	Oak chips 125 °C	2.5	5.1	277.9 ± 5.7
*Castanea sativa*	Chestnut chips 175 °C	2.6	5.2	533.1 ± 7.4
	Chestnut chips 125 °C	2.1	4.2	551.6 ± 27.4
*Robinia pseudoacacia*	Locust chips 175 °C	1.6	3.1	340.5 ± 4.2
	Locust chips 125 °C	1.1	2.1	320.6 ± 4.8
**Vine By-Products**				
*Vitis vinifera*	Vine canes 175 °C	5.3	10.5	94.2 ± 1.7
	Vine canes 125 °C	5.7	11.3	91.7 ± 3.7
	Vine roots 175 °C	7.1	14.1	120.9 ± 2.7
	Vine roots 125 °C	1.9	3.8	65.6 ± 1.7

^1^ Extraction yield % = weight of extracted plant residues/weight of plant raw sample × 100; ^2^ Concentration: g of dry matter in one-liter of natural extract.

**Table 2:** Phenolic compounds identified in the active maritime pine sawdust extract obtained at 175 °C.

Compounds	Retention Time (min)	λmax (nm)	(M − H)^−^	Concentration (mg g^−1^ of Extract)
Phenolic Acids/Aldehydes/Alcohols				
Protocatechuic acid	1.8	260	153	0.25 (± 0.01)
Vanillic acid	3.7	262	167	0.53 (± 0.06)
Caffeic acid	3.8	325	179	0.15 (± 0.01)
Coniferyl alcohol	4.6	264	179	4.89 (± 0.22)
Vanillin	4.7	280	151	0.64 (± 0.01)
Ferulic acid	5.1	325	193	0.35 (± 0.01)
Coniferyl aldehyde	5.9	340	177	0.83 (± 0.01)
Unknown ^1^	10.6	253	329	1.90 (± 0.06)
**Lignans**				
Nortrachelogenin	6.9	281	373	7.30 (± 0.25)
Pinoresinol ^2^	7.7	281	357	2.84 (± 0.29)
**Flavonoids**				
Pinobanksin ^3^	9.2	289	271	1.01 (± 0.01)
Pinocembrin	11.8	289	255	0.41 (± 0.02)
**Total**				21.09 (± 0.40)

^1^ Quantified using coniferyl alcohol as standard. ^2^ Quantified using nortrachelogenin as standard. ^3^ Quantified using pinocembrin as standard. Standard deviation using three replicates in brackets.

**Table 3:** Origin and characteristics of *Fusarium graminearum* strains used in this study.

Strain	Source	Host	Country	Chemotype
Fg 605	INRAE MycSA collection, France ^1^	Unknown	Unknown	DON/15ADON
Fg 156	INRAE MycSA collection, France ^1^	Wheat	France	DON/15ADON
Fg 164	INRAE MycSA collection, France ^1^	Wheat	France	DON/15ADON
Fg 91	INRAE MycSA collection, France ^1^	Corn	France	NIV/FX
34W23.4F9	AgResearch, Hamilton, New Zealand [48]	Unknown	New Zealand	DON/15ADON
PH-1	Fungal Genetic Stock Center, USA	Corn	USA	DON/15ADON
CBS 185.32	Westerdijk Fungal Biodiversity Institute, The Netherlands ^2^	Corn	Unknown	DON/15ADON

^1^ INRAE MycSA strains are stored in the International Center for Microbial Resources-Filamentous Fungi, Marseille, France (https://www6.inrae.fr/cirm_eng/Filamentous-Fungi/Strains-catalogue). ^2^ Westerdijk Fungal Biodiversity Institute, previously Centraalbureau voor Schimmelcultures (CBS). Fg, *Fusarium graminearum*; DON, deoxynivalenol; 15ADON, 15-acetyl-4-deoxynivalenol; NIV, nivalenol; FX, fusarenon X.

## Data Availability

The data presented in this study are available on request from the corresponding author.

## Sample Availability

Not available.
